# Schwannoma of the breast: Two cases report and a literature review

**DOI:** 10.1097/MD.0000000000043011

**Published:** 2025-06-20

**Authors:** Sen Li, Yingfang Shi, Zhichun Wang

**Affiliations:** a Breast Surgery, Jiujiang City Key Laboratory of Cell Therapy, JiuJiang No. 1 People’s Hospital, Jiangxi Province, Jiujiang, PR China.

**Keywords:** S-100 protein, schwannoma of the breast, surgery

## Abstract

**Rationale:**

Schwannoma, a benign tumor originating from Schwann cells, is a rare entity in the breast. While most schwannomas are found in peripheral nerves of the neck, buttocks, forearms, and lower limbs, breast schwannomas are exceedingly uncommon, often leading to diagnostic challenges.

**Patient concerns:**

Two middle aged women presented with palpable breast lumps. Both patients were concerned about the mass and opted for surgical excision due to financial constraints and reluctance to undergo a core needle biopsy.

**Diagnoses:**

Clinical examination, imaging studies (ultrasound and mammography), and histopathological analysis confirmed the diagnosis of breast schwannoma in both cases. Immunohistochemistry showed positive staining for S-100 protein and other markers, confirming the benign nature of the tumors.

**Interventions:**

Both patients underwent lumpectomy under local anesthesia. Surgical resection was performed with complete excision of the mass and surrounding normal tissue.

**Outcomes:**

Histopathological examination post-surgery confirmed the diagnosis of schwannoma for both cases. No postoperative complications were noted, and both patients showed a favorable recovery, with no signs of recurrence during follow-up.

**Lessons:**

Breast schwannomas, although benign, are rare and can be easily misdiagnosed as other.

## 1. Introduction

Schwannoma, a benign tumor originating from Schwann cells, also referred to as neurilemmoma, is a rare entity that can manifest in various locations throughout the human body. While most schwannomas arise from peripheral nerves in regions such as the neck, buttocks, forearms, and lower limbs, occurrence within the breast is notably infrequent. Reported cases of breast schwannoma are sparse in the literature, underscoring the rarity of this condition.

Schwannomas in the breast can occur across all age groups, though they are most commonly observed in individuals between 20 and 50 years old. Notably, Das Gupta et al reported that breast neurilemmoma accounted for a mere 2.6% of all reported neurilemmomas, indicating its rarity compared to occurrences in other anatomical sites.^[1]^

Clinical presentation of breast schwannomas varies depending on tumor size and location. While smaller lesions may remain asymptomatic, larger tumors have the potential to elicit symptoms such as pain or paralysis, often resulting from nerve compression. Characteristically, breast schwannomas tend to exhibit benign features, presenting as solitary masses of varying size with slow growth kinetics. Upon palpation, these masses typically display an oval or fusiform shape, with a distinct texture, clear boundary, and smooth surface. Larger tumors may manifest with hemorrhage, mucinous changes, or cystic alterations.

In this paper, we present 2 cases of breast schwannoma diagnosed and managed at our institution. Through a comprehensive analysis of the patients’ clinical histories, imaging findings, surgical interventions, and postoperative outcomes, we aim to contribute to the existing body of literature on this rare pathology. By providing detailed insights into the clinical course and management strategies employed in these cases, we seek to enhance understanding and facilitate accurate diagnosis and treatment of breast schwannoma. Written informed consent was obtained from both patients.

## 2. Cases presentation

Case 1: A early 50s female presented with a complaint of a right breast mass during a physical examination 2 weeks prior, without associated symptoms such as tenderness, redness, swelling, elevated skin temperature, fever, fatigue, or nipple discharge. Upon admission to the breast outpatient clinic of our hospital on May 25, 2023. On physical examination, a palpable mass measuring 3.0 cm × 2.0 cm was identified at 3.0 cm from the nipple at 8 o’clock in the right breast, characterized by a tough texture, smooth surface, clear boundary, and good mobility. No enlarged lymph nodes were noted in the bilateral axillas.

**Figure 1.**
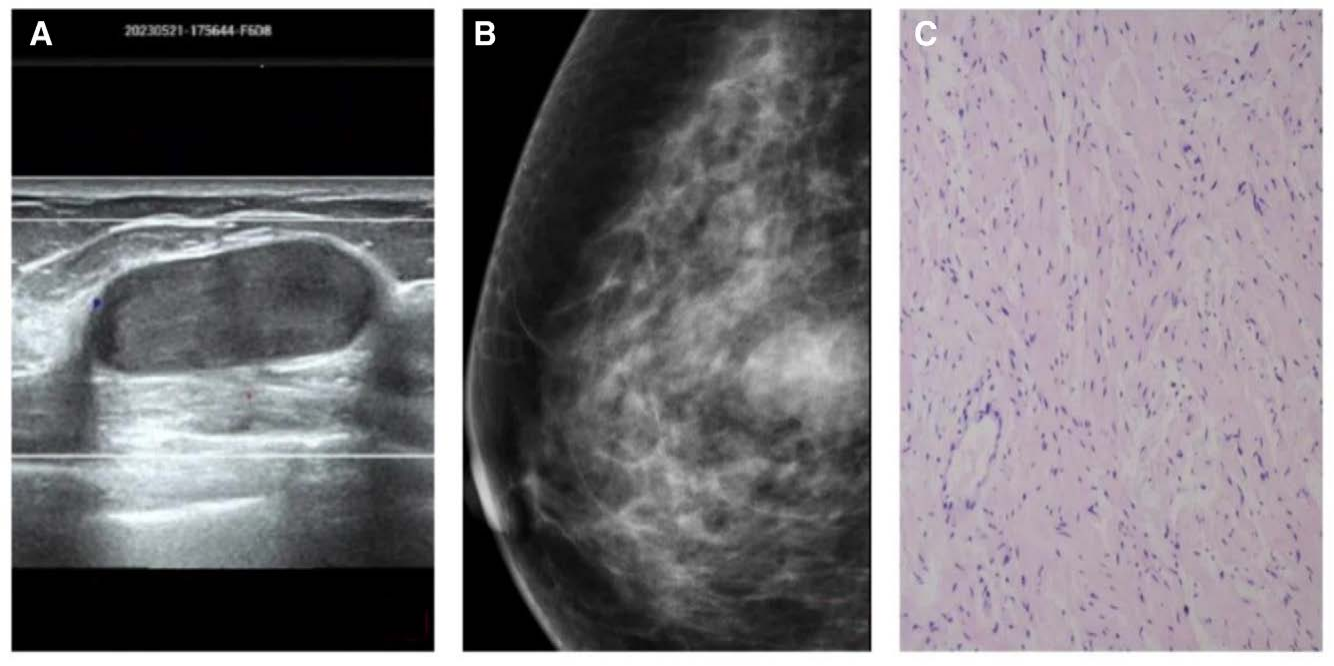
(A) Ultrasound image of the right breast showing an oval and hypoecoic mass, with circumscribed margins and posterior enhancement. (B) Mammography image showing showing a noncalcified circumscribed oval mass. (C) (Hematoxylin and eosin [HE] × 100) tumors were dominated by spindle cells with more obvious atypia and normal reshaped squamous epithelium. HE = hematoxylin and eosin.

Breast ultrasound indicates a low level of suspicion for malignancy (>2%–≤10%) in the middle and posterior region of the right breast (breast imaging-reporting and data system 4A class; Fig. 1A). Outpatient mammography also revealed a low-density mass in the right breast (breast imaging-reporting and data system 4A class), prompting admission to the hospital for further evaluation (Fig. 1B).

Following meticulous preoperative preparation, the patient underwent resection of the right breast lesion under local anesthesia. Intraoperatively, a mass approximately 3.0 cm × 1.5 cm in size was observed in the right breast, exhibiting an unclear boundary and obvious capsule, with a gray-white solid cut surface resembling fish flesh. Complete excision of the mass and surrounding normal glandular tissue was performed, and the specimens were sent for pathological examination. Histopathological evaluation confirmed the diagnosis of schwannoma in the right breast. Immunohistochemical analysis revealed negative staining for beta-catenin and estrogen receptor, positive staining for retinoblastoma protein, S-100 protein, and vimentin, among other markers (Fig. 1C).

**Figure 2.**
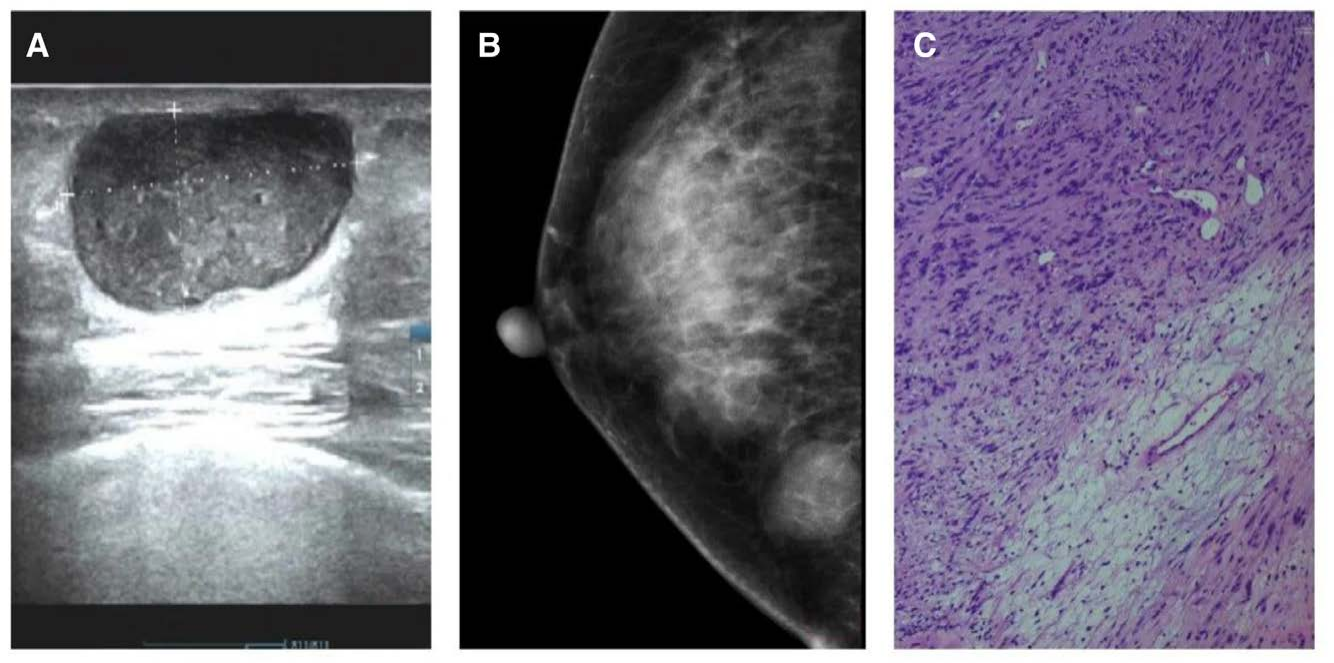
(A) Ultrasound image of the right breast showing a subcutaneous oval, hypoecoic and heterogenous mass, with circumscribed margins and posterior enhancement. (B) Mammography image showing a noncalcified circumscribed oval mass in the lower inner quadrant of the right breast. (C) (Hematoxylin and eosin [HE] × 20) S-100 was expressed clearly and strongly in atypical cells. HE = hematoxylin and eosin.

Case 2: A early 40s female presented to the hospital with a lump in her right breast of 1 month’s duration. Physical examination revealed no palpable enlargement of the superficial lymph nodes, nor signs of skin redness, swelling, elevated skin temperature, orange peel change, or desquamation of the areola. Palpation of the right breast identified a mass measuring approximately 2.0 cm × 3.0 cm at 6 o’clock, 2 cm from the nipple. The mass was described as leathery, clear, smooth, mobile, and non-tender. No palpable mass was appreciated in the left breast (Fig. 2A).

Ultrasonography of the right breast revealed a subcutaneous, oval, hypoecoic and heterogenous mass, with circumscribed margins and posterior enhancement. Mamography confirmed the presence of a noncalcified circumscribed oval mass in the lower inner quadrant. These imaging findings were consistent with a breast imaging-reporting and data system 4A. On June 2, 2023, the patient underwent resection of the right breast lesion under local anesthesia. Intraoperative examination revealed a mass measuring approximately 2.5 cm × 2.0 cm, exhibiting unclear boundaries and an obvious capsule, with a gray and yellow solid cut surface. Complete excision of the tumor and surrounding normal glandular tissue was performed (Fig. 2B).

Subsequent pathological analysis confirmed the diagnosis of neurilemmoma in the right breast. Immunohistochemistry demonstrated wild type tumor protein p53, low Ki-67 antigen proliferation index, negative staining for signal transducer and activator of transcription 6, desmin, and smooth muscle actin, among other markers, with positive staining for SRY-box transcription factor 10 and vimentin (Fig. 2C).

## 3. Disscusion

Schwannomas, originating from Schwann cells, are among the common types of peripheral nerve sheath tumors. They typically occur in young adults around the age of 30 years and are exceedingly rare in children.^[2]^ Collins et al were the first to report breast schwannomas.^[3]^ These tumors represent only about 2%–3% of all schwannomas, in contrast to approximately 0.2% of all breast tumors. Interestingly, the incidence of breast schwannoma in males and females is nearly equal, with documented cases in both sexes.^[4–6]^ Most other breast tumors tend to occur in the outer upper quadrant, whereas schwannomas can affect all quadrants simultaneously.^[1]^ Due to their low incidence in the breast, there are limited reports regarding the ultrasound and mammography findings of these tumors.

Schwannomas typically present with clinical and imaging findings resembling those of benign tumors.^[7]^ Ultrasound plays a crucial role in distinguishing between benign and malignant breast schwannomas by assessing the clarity of the tumor capsule and its shape. Ultrasonographic features of breast schwannomas include a clear and round tumor boundary, internal hypoechoic characteristics, posterior enhancement, and rich blood flow signals. Cystic or hemorrhagic masses may exhibit irregular anechoic areas with uneven internal echoes and rich blood flow signals. High frequency mammography often reveals round-like dense shadows with well-defined margins, coarse granular calcification spots within the tumor, relatively rich surrounding blood supply, and vague subcutaneous fat layers. Axillary lymph node enlargement is typically absent.^[7–10]^

The diagnosis of schwannoma relies primarily on histopathological examination. Typically, schwannomas present as round or lobulated tumors with a gray-white, solid, fish-like appearance and a distinct capsule upon cut surface examination. Microscopically, schwannomas are characterized by spindle cell morphology, with tumor cells filling the entire background. These cells are predominantly oval or spindle-shaped and exhibit relatively uniform features.^[11]^ Histologically, schwannomas exhibit distinct Antoni A and Antoni B zones. The Antoni A zone comprises fusiform Schwann cells with eosinophilic cytoplasm and basophilic nuclei arranged in a palisade pattern within a collagen matrix. These cells often form parallel arrangements with eosinophilic cytoplasmic processes known as Verocay bodies.^[2,10]^ In contrast, the Antoni B zone contains fewer cells with loose stroma and myxoid changes, along with increased vascularity and infiltration of inflammatory cells.^[12]^ During tumor progression, there may be a transition from the Antoni A to Antoni B region.^[13]^ Immunohistochemically, schwannoma cells typically stain positive for S-100 protein, which serves as a reliable marker for identifying these tumors.

Benign breast schwannomas typically respond well to surgical resection, offering a favorable prognosis. However, there exists a risk of malignant transformation, underscoring the importance of postoperative monitoring. Shuayb et al proposed a treatment regimen for malignant neurilemmomas of the breast involving total mastectomy followed by adjuvant chemotherapy and radiotherapy, necessitating long-term surveillance.^[14]^ Malignant breast schwannomas commonly recur locally postoperatively, with distant metastasis primarily involving hematogenous spread to the lungs. Lymph node metastasis is infrequent. The 5-year survival rate for malignant breast schwannoma is approximately 20%, with some cases demonstrating survival up to 13 years.^[14]^ Notably, lung metastasis is the leading cause of mortality. For benign breast schwannomas, surgical resection remains the primary therapeutic modality, with a favorable prognosis. Optimal management involves complete surgical excision while preserving maximal residual nerve function. Conversely, malignant peripheral nerve sheath tumors necessitate surgical resection with adequate margins.^[15]^

Breast schwannomas, although benign, are infrequent and frequently misidentified as other breast lesions, including fibroadenomas, phyllodes tumors, mesenchymal tumors, and even breast cancers. Despite their benign nature, individuals with neurofibromas face an elevated risk of malignant transformation within schwannomas. Consequently, further clinical research and exploration are warranted to enhance the diagnosis and treatment of breast schwannomas.

## Author contributions

**Conceptualization:** Zhichun Wang.

**Investigation:** Yingfang Shi.

**Supervision:** Yingfang Shi.

**Writing – original draft:** Sen Li.

**Writing – review & editing:** Zhichun Wang.
